# Establishing the financial burden of alopecia areata and its predictors

**DOI:** 10.1002/ski2.301

**Published:** 2023-10-15

**Authors:** Fabio Zucchelli, Matthew Harries, Andrew Messenger, Kerry Montgomery

**Affiliations:** ^1^ Centre for Appearance Research University West of the England Bristol UK; ^2^ Salford Royal Hospital Northern Care Alliance NHS Foundation Trust Manchester Academic Health Science Centre Manchester UK; ^3^ Centre for Dermatology Research Faculty of Biology, Medicine and Health University of Manchester Manchester UK; ^4^ University of Sheffield Sheffield UK; ^5^ Alopecia UK Shipley UK

## Abstract

**Background:**

Alopecia areata (AA) can have a significant impact on wellbeing. Consequently, individuals with AA often seek treatments or products to promote hair regrowth or camouflage their hair loss that incur a financial cost to the individual.

**Objectives:**

The current study aimed to examine the direct financial burden of AA to understand the wider impact of the condition and the factors which influence spending on products and services.

**Methods:**

A total of 829 United Kingdom based participants completed an online survey. Demographic and condition‐specific data were collected, alongside spending on AA‐related products and services. Participants were asked about their use of products and services, the associated costs, how they financed these costs, and their household income to determine what percentage of income they spent on products and services.

**Results:**

Participants predominantly identified as female (85.9%), white (92.7%) with a mean age of 42.7 years and a median AA duration of 10.94 years. Female gender, Asian ethnicity, lower income, and worse AA symptoms predicted higher spend from income. Wigs were the most common product used and incurred the greatest cost (median £700). The highest cost for men was private dermatology services (median = £550). On average people spent 3% of their disposable income (prior to housing costs) on AA‐related products and services.

**Conclusions:**

This study outlines the risk factors associated with higher financial burden from managing AA which require consideration by health providers, commissioners, and policy makers when designing services to support the wellbeing of people living with AA.

## Graphical abstract

This study investigated the financial burden of alopecia areata (AA) and its influencing factors on spending among 829 participants in the UK. Female gender, Asian ethnicity, lower income, and more severe AA symptoms were associated with higher expenditure on AA‐related products and services. These findings underscore the importance of considering these risk factors when developing support services for individuals with AA.

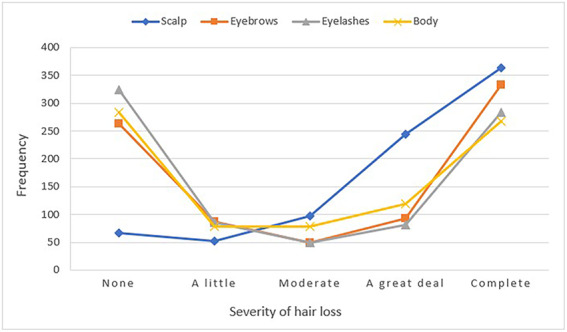

**What is already known about this topic?**

- Alopecia areata (AA) has a significant impact on psychosocial wellbeing and increased health service utilisation.
- In the absence of effective treatments individuals use strategies to camouflage hair loss.

**What does this study add?**

- AA can have significant financial implications with individuals spending on average 3% of their disposable income on products/services.
- Female gender, Asian ethnicity, worse AA symptoms and lower income predict higher financial burden.
- Men used comparably fewer aesthetic products but accessed products and services aimed at treatment and regrowth at similar rates to women.

## 1 INTRODUCTION

Alopecia areata is a follicular inflammatory condition causing hair loss, with a peak onset of age 25–29 years^1^ and a lifetime prevalence of approximately 2%.^2^ Hair loss from AA typically begins with small patches on the scalp but can progress to its more severe forms involving total scalp (alopecia totalis) and body (alopecia universalis) hair loss. Living with AA can also have deleterious consequences on psychosocial wellbeing, owing predominantly to its visible impact on appearance.^3^

To manage the change in appearance, many use strategies to conceal or camouflage their hair loss. Wigs, for example, can reduce fear of negative evaluation from others and improve social confidence,^4^ and can therefore be considered an orthotic for those choosing to use them.^5^ However, wigs vary greatly in their quality, with higher quality often incurring higher costs in the United Kingdom (UK) where sporadic wig provision exists across National Health Service (NHS) localities.^5^ Other, more emerging aesthetic methods such as medical tattooing to camouflage hair loss through pigment (“microblading”), tattoo ink or permanent make‐up to restore the look of eyebrows are also growing in popularity.^6^

While promising treatments may be on the horizon for AA,^7^, ^8^, ^9^ the efficacy of current treatments is limited.^10^ Along with the disparity in treatment options between hospitals and long NHS dermatology waiting times, this means people with AA resort to private dermatology services and alternative remedies such as non‐prescribed shampoos. However, we currently have a very limited understanding of what products and services people living with AA are using to manage their appearance and target hair regrowth, as well individuals' perception of their effects on quality of life. Strategies for managing AA may also differ by gender, with men who have AA noting sociocultural barriers to using many aesthetic products like wigs.^11^

Despite most of these products and services also incurring monetary costs, no research has quantified their direct financial cost. Recent case‐control research established the notable indirect costs of AA via elevated rates of mental health service use, diagnoses of common mental health conditions, absenteeism and unemployment in affected individuals compared to controls.^12^, ^13^ Adding an understanding of the direct financial burden of AA, as well as how individuals manage these costs, would complete the picture of how the condition impacts individuals socioeconomically. Research with people living with AA in the United States indicates at least a moderate financial burden on the individual on average, elevated pharmaceutical expenses, and significant costs for using wigs.^13^, ^14^, ^15^ However, differences in health systems and socioeconomic climates limit the transferability of these findings to other countries like the UK.

The chronic nature of AA also increases the likely personal cost. It is therefore important to move beyond methodologies relying on healthcare databases (e.g.,^13^, ^14^, ^15^) that include people with AA at and around the point of diagnosis, to those who have been managing AA over many years. In a qualitative study of people who had been diagnosed with AA 15 years ago on average, researchers reported a “costs of concealment” subtheme, which as well as describing emotional, functional and social costs, captured participants' perceived financial costs, which some described as increasing over the years from trying out various products and services, often emerging through new technology.^16^ It would therefore be worth investigating whether AA duration predicts direct financial burden of managing the condition.

It is also important to understand which factors, if any, predict greater financial burden for people with AA. Research suggests that female gender, older age and white ethnicity are associated with worse mental health outcomes in those diagnosed with AA, while the relationship between symptom severity and mental health is unclear.^12^ Compounding compromised mental health with greater financial burden would clearly alert cause for concern given the intertwined relationship between the two outcomes.^17^ Relatedly, the extent to which spending on AA is proportionate to one's means is currently unknown.

This study's aims are threefold:

1. Measure the scope and frequency of products and services used for managing AA, and their overall perceived utility in enhancing quality of life.
2. Quantify the direct financial burden of using these products and services, and understand how individuals manage this burden.
3. Examine demographic and disease‐related factors as potential predictors of financial burden from managing AA.

## 2 PATIENTS AND METHODS

### 2.1 Study design and setting

This study was conducted via an online survey using Qualtrics. The research team involved a collaboration between a national alopecia charity, Alopecia UK, and an academic partner. The project objectives and consequent survey encompassed socioeconomic and psychological outcomes, so this article presents only those relevant to financial burden.

We utilised patient involvement (PI) during the study design phase via advisory group meetings and survey feedback to maximise the study's acceptability and applicability.^18^ After confirming consent, participants provided demographic data, which included gross household income band and composition to enable estimates of financial burden from AA costs. For AA status, participants reported their disease duration, and completed the AA Symptom Impact Scale—Severity Subscale (AASIS‐S^19^), in which they rated the severity of seven symptoms from 0 (not present) to 10 (as bad as you can imagine) over the preceding week. In this study the AASIS‐S displayed acceptable reliability (*α* = 0.7). Participants also completed the AA Patient Priority Outcomes—Hair Loss items (AAPPO‐HL^20^), by rating the current severity of their hair loss separately on their scalp, eyebrows, eyelashes and body, using a five‐point scale from 0 (no hair loss) to 4 (complete hair loss).

Participants were presented with an itemised list of products and services related to AA, devised through PI. They were asked to select which they had used over the preceding 12 months, as well as between diagnosis and before the preceding 12 months. They were also asked to estimate their itemised spend over the preceding 12 months. A single item scale was created for this study asking participants to select the statement that best characterised their perceived quality of life impact from the products and services they had paid for during the preceding 12 months, ranging from 1 (“made things much worse”) to 5 (“made things much better”). We also asked participants to select all statements which applied to describe how they managed the AA‐related costs they had incurred, for example, ‘I have used my own savings’.

Data collection took place between May and August 2022. Recruitment was predominantly conducted online via Alopecia UK's online distribution channels. This included targeted study adverts seeking typically underrepresented group in AA research, namely men and individuals from black and Asian ethnic minority groups.

### 2.2 Patients

To be eligible, respondents had to self‐report a confirmed diagnosis of AA (including alopecia totalis and universalis) by a general practitioner or dermatologist, be aged 16+, and live in the UK. We targeted a final sample size of *n* ≥ 665, applying Cochran's (1977) formula for calculating minimum sample sizes when the population to which the sample seeks to generalise is known,^21^ as is the case for people with AA in the UK.^1^

### 2.3 Statistical analyses

All data were processed and analysed by the first author using SPSS version 28.0. Missing data at the person, item and variable level were handled according to published guidance.^22^ For continuous outcome variables with >5% missingness, Little's Test indicated data were Missing Completely at Random (*X* ^2^ (203) = 220.32, *p* = 0.19) so we applied five multiple imputations and reported results from the pooled dataset.

The first two study aims were addressed using descriptive analyses, including frequencies, percentages, medians, and interquartile ranges (where data were non‐normally distributed) and were split by gender where relevant. To quantify financial burden from AA, we summed participants' itemised costs for the preceding 12 months at the individual level to provide a total cost. We also calculated this cost as a percentage of participants' equivalised disposable household income (hereafter “income”) to estimate financial burden. To calculate this income, participants' gross income was converted to disposable income (pre‐housing costs) using UK population data by income band.^23^ Equivalisation was conducted using the modified OECD equivalence scale^24^ to account for household composition and estimated financial responsibility.

Hierarchical linear regression was used to address the third aim, with absolute cost as a percentage of income as the outcome variable. The hierarchical approach facilitated comparison between demographics, symptom severity, AA duration and income level as separate factors to explain variance in financial burden, while also assessing the unique predictive strength of each variable. Before conducting the analysis, data were checked against assumptions. Multicollinearity was found between the eyebrow and eyelash items of the AAPPO‐HL, so these were combined into a single hair loss variable for analysis.

## 3 RESULTS

### 3.1 Participant characteristics

**FIGURE 1:**
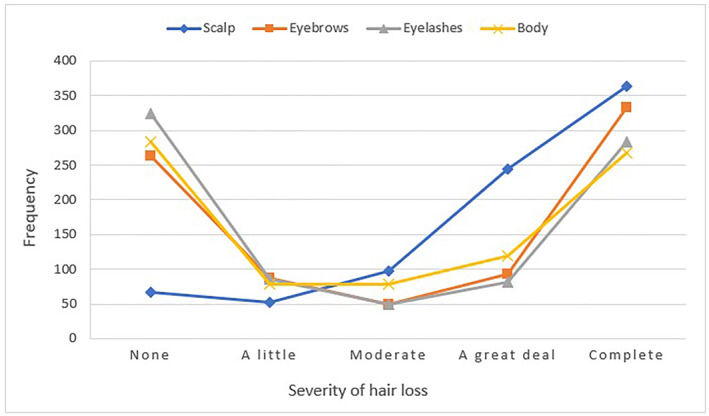
AAPPO‐HL severity frequencies by hair loss site (*n* = 826).

The final sample comprised 829 full and partial respondents. The sample size varied by question, so *n* for each question is presented in the results below. Table 1 shows participant characteristics. The majority of the sample were female and white, the mean age was 42 and participants had been living with AA for a median of approximately 11 years. To highlight the mixed distribution patterns across site‐by‐site current hair loss severity scores (measured by AAPPO‐HL), these frequencies are displayed in Figure 1. It shows just under half reported complete scalp hair loss, and most participants had either complete hair loss or none on their eyelash, eyebrows or body.

**TABLE 1:** Participant characteristics.

	*n*
Gender (*n* = 827)	
Female	711 (85.9%)
Male	114 (13.8%)
Non‐binary/third gender	2 (0.2%)
Age (mean and SD) (*n* = 827)	42.72 (±13.78)
Ethnicity (*n* = 822)	
White	762 (92.7%)
Asian/Asian British	23 (2.8%)
Mixed	22 (2.7%)
Black/Black British	10 (1.2%)
Other	5 (0.6%)
Relationship status (*n* = 820)	
Married/in civil partnership	373 (45.5%)
In a relationship	215 (26.2%)
Single/dating	172 (21.0%)
Separated/divorced/widowed	59 (7.2%)
Income^a^ (median and interquartile range) (*n* = 726)	£26,297.39 (£19,455.34)
AA duration (median and interquartile range) (*n* = 824)	10.94 years (21.1 years)
AASIS‐S symptom severity^b^ (mean and SD) (*n* = 828)	4.53 (±2.10)

^a^ Equivalised disposable household income.

^b^ Multiple imputation applied to variable.

### 3.2 AA‐related products and services usage

Table 2 shows participants' usage of products and services to manage AA (a) over the preceding 12 months and (b) after diagnosis and before the preceding 12 months. Usage rates are also split by gender, showing that men used fewer products except hats, non‐prescribed shampoos and other non‐prescribed scalp treatments, and that men and women used AA‐related services at a comparable rate.

**TABLE 2:** Usage of alopecia areata products and services.

	After diagnosis and before preceding 12 months^a^			During preceding 12 months		
	Frequency of usage from respondents (from *n* = 761) (%)	% of females using item (from *n* = 653)	% of males using item (from *n* = 105)	Frequency of usage from respondents (from *n* below) (%)	% of females using item (from *n* = 663–677)	% of males using item (from *n* = 102–107)
Products involving services						
Wigs and toppers (prescribed or non‐prescribed)	502 (66%)	75.7%	5.7%	449/788 (56.9%)	65.9%	0.9%
Microblading/Eyebrow tattoo/Permanent make‐up	263 (34.6%)	38.6%	7.6%	248/778 (31.9%)	35.3%	9.3%
Hair system	76 (10%)	11%	0%	44/782 (5.6%)	6.4%	0.9%
False eyelashes by a professional	50 (6.6%)	7.5%	1%	28/778 (3.6%)	4.3%	0%
Scalp micropigmentation/Head tattoo	19 (2.5%)	2.3%	2.9%	22/782 (2.8%)	3%	1.9%
Products						
Hats	431 (56.6%)	57.7%	49.5%	349/782 (44.6%)	44.6%	45.4%
Vitamins or supplements for hair growth	430 (56.5%)	59.4%	38%	359/775 (46.3%)	48%	38.3%
Eyebrow pencils	401 (52.7%)	59.6%	9.5%	308/778 (39.6%)	45.1%	6.5%
Head scarfs	374 (49.1%)	55.4%	9.5%	301/782 (38.5%)	43.5%	6.5%
Non‐prescribed shampoos for hair growth	347 (45.6%)	47.2%	36.2%	255/775 (32.9%)	33.8%	37.3%
False eyelashes—self‐applied	280 (36.8%)	7.5%	1.9%	252/778 (32.4%)	37.1%	1.9%
Any other non‐prescribed scalp treatments	212 (27.9%)	28.9%	21%	151/775 (19.4%)	19.4%	20.6%
Temporary eyebrow transfers/Stick‐ons	154 (20.2%)	21.9%	8.6%	110/778 (14.1%)	15.6%	4.7%
Services						
Private dermatologist	133 (17.5%)	17.8%	15.2%	70/767 (9.1%)	8.5%	13.2%
Private mental health practitioner	83 (10.9%)	11.6%	6.7%	96/740 (13%)	12.6%	13.1%
Alternative therapies (e.g. reiki, reflexology, nutritionist)	144 (18.9%)	20.4%	9.5%	131/775 (16.9%)	11.9%	3.7%
Private hair clinic^b^	132 (17.3%)	18.2%	11.4%	64/771 (8.3%)	7.5%	9.3%

^a^ Median duration = 10.3 years.

^b^ Non‐medically qualified.

Asked about the quality of life impact of the products and services used by participants over the preceding 12 months, the most selected statement was ‘Made things a little better’ (228/643; 35.5%), followed by ‘Made no difference’ (220/643; 34.2%), and ‘Made things much better’ (131/643; 20.4%). Ten per cent agreed that the products and services had made things much or a little worse.

### 3.3 Financial burden of AA

After summing all products and services used by participants over the preceding 12 months for AA—including individuals who had not spent money on these items—we applied multiple imputation to the resultant variable of total cost, leading to *n* = 828. The median total cost was £801.50 (quartile boundaries = £206 and £1946.55). The median total cost for women was £930, with quartile boundaries of £300 and £2125.40. For men, this was lower at £155, with quartile boundaries of £18.75 and £696.80.

Within the subsample of those who had spent on products and services over the preceding 12 months, wigs were the most commonly used product or service, incurring a median annual cost for women of £700 (interquartile range = £1300). The second highest cost came from combined eyebrow and eyelash products, with women spending a median of £200 (interquartile range = £310). Although only 14 of 107 men spent money on private dermatology services, this represented men's biggest expense (median = £550; interquartile range = £1167.50). Table S1 shows a cost breakdown for all product/services.

The median percentage of participants' absolute cost as a function of income (*n* = 732) was 3.0%, with quartile boundaries of 0.8% and 7.3%. For women the median was 3.6% (quartile boundaries of 1.2% and 8.3%), and for men it was 0.3% (quartile boundaries of 0% and 2.4%).

**FIGURE 2:**
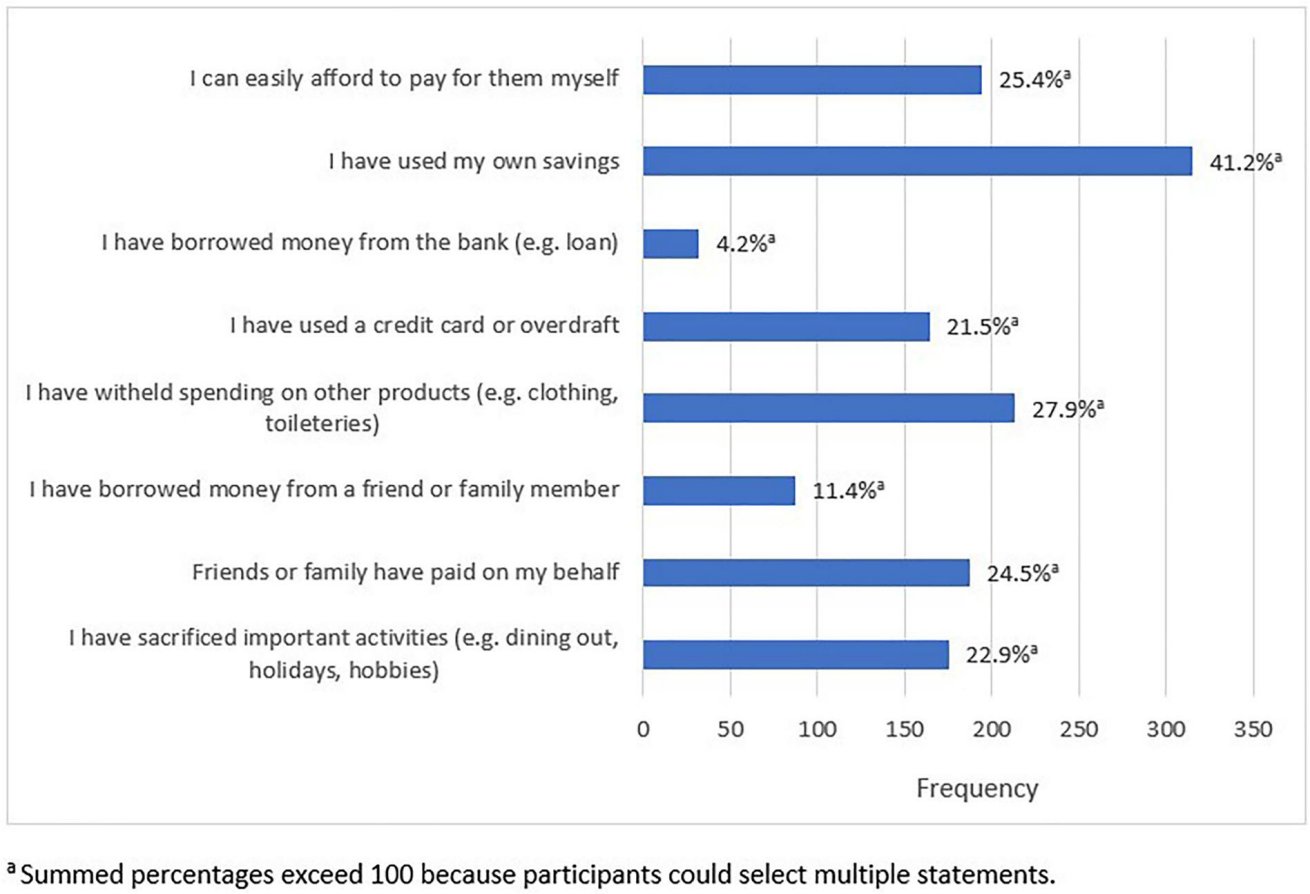
Participants' strategies for managing AA‐related costs (*n* = 764).

Figure 2 displays the frequencies of participants' selected statements regarding their strategies for managing their AA‐associated costs. The most commonly selected statement was ‘I have used my own savings’ (∼40%), and the least selected statement was ‘I have borrowed money from the bank (e.g. loan)’ (∼4%).

### 3.4 Predictors of financial burden

Table 3 shows the results of the hierarchical regression analysis with financial burden as outcome, and candidate predictors of financial burden entered into the analysis in four steps. The results are taken from the imputed dataset (*n* = 724). In step 1, demographics combined explained 3% of variance in financial burden. Female gender uniquely predicted greater financial burden, as did Asian ethnicity and younger age until the latter's effect was suppressed by disease duration and income level being added to the model. At step 2, greater AA severity collectively explained 5% of variance in financial burden, within which only symptom severity was a unique predictor. In step 4, lower income also explained a significant amount of variation (6%) in financial burden.

**TABLE 3:** Hierarchical regression analysis for financial burden (spend as % of income).

	*R* ^2^	∆*R* ^2^	*F*	*p*	*B* ^a^	*SE B* ^a^	*β* ^a^
Step 1	0.031	0.031	4.53	<0.001*			
Gender^b^				0.103	−3.33	1.13	−0.11
Age				0.003*	−0.05	0.03	−0.06
Ethnicity: Asian/Asian British^c^				0.035*^d^	5.30	2.51	0.06
Ethnicity: Black/Black British^c^				0.245	−3.96	3.41	0.04
Ethnicity: Mixed^c^				0.852	−0.43	2.31	0.01
Step 2	0.081	0.050	9.24	<0.001**			
Symptom severity (AASIS‐S)				<0.001**	1.00	0.22	0.20
Scalp hair loss severity (AAPPO‐HL)				0.593	0.26	0.49	0.03
Eyelash & eyebrow hair loss severity (AAPPO‐HL)				0.148	−0.67	0.47	−0.11
Body hair loss severity (AAPPO‐HL)				0.054	0.92	0.48	0.15
Step 3	0.082	0.001	0.55	0.458			
Disease duration (years)				0.271	−0.03	0.03	−0.05
Step 4	0.144	0.062	51.92	<0.001*			
Income level (£)				<0.001*	0.00	3.00	−0.25

^a^ Value taken from variable alongside all predictors in Step 4 model.

^b^ Female gender as reference category.

^c^ White ethnicity as reference category.

^d^ Only significant using imputed dataset.

*p < 0.05; **p < 0.001.

## 4 DISCUSSION

To our knowledge, this is the first study to examine the direct financial impact of AA for UK‐based individuals. Recruiting participants living with AA for a decade on average also facilitated a stronger understanding of the longer‐term costs associated with AA‐related products and services. In the predominantly female sample, wigs were the most popular and expensive product since diagnosis and over the preceding year. This reinforces the call for equity and quality of wig provision across the UK, as per the Charter for Best Practice for NHS Wig Provision.^5^

Given the costs of wigs outlined in the study it is unsurprising that women experienced a higher financial burden from AA. This is concerning alongside recent findings suggesting UK women recently diagnosed with AA experience worse mental health outcomes compared to men.^12^ Although challenging to interpret the annual median 3% (£800) spend on AA‐related products and services from income in terms of affordability, for context, this is equivalent to a third of the current UK average household cost of energy (£2,500^25^), amid rising living costs. Of note, more participants had drawn from their savings and withheld spending on other products than those who stated they could afford the costs.

In addition to female gender, participants with worse AA symptoms, lower income and Asian ethnicity were also more likely to spend a greater proportion of their income on products and services. It is noteworthy that global AA symptom severity significantly predicted financial burden while site‐specific hair loss did not. The presence of typical physical symptoms beyond hair loss listed in the global severity measure, such as itching, may infer a preference towards costlier products such as human hair wigs (as opposed to acrylic alternatives) to minimise discomfort. It could also be that individuals typically purchase aesthetic products and services only once scalp hair loss has reached a moderate extent, leading spending to plateau between moderate and severe hair loss and hence to be indiscernible in the regression model.

The finding that lower income predicted greater financial burden of AA suggests that those with lower means may be spending similar amounts to those with higher means, and hence a higher proportion of income. This suggests that spending on products and services for AA is considered essential, regardless of disposable income level. This supports the notion of wigs as a necessary orthotic for daily functioning.^5^ Asian ethnicity being associated with greater spending is an interesting finding, as there are fewer wigs and products available that target Asian hair specifically, and these specialist products can be more expensive. However, there were disproportionately few Asian participants, meaning this should be interpreted as an important signal for future research rather than a robust finding.

That disease duration did not predict financial burden is also noteworthy. This may reflect a prominence of individual difference factors in coping over time. Two opposing forces reported in the literature may also nullify each other statistically: Although more products become available over time due to technological advances,^16^ the early stages after AA diagnosis are characterised by a desire for concealment of hair loss for many, followed by greater acceptance and hence a waning demand for products and services.^26^

Participants' perceived impact on quality of life conferred by products and services broadly pointed to perceived value. However, with a third stating that they had made no difference, and 1 in 10 even stating they had some negative impact, this suggested mixed experiences across the sample. In the case of the most popular product for women, wigs, the variability in quality and affordable access to higher quality wigs may partly explain negative experiences, as well as high costs associated with wigs. For example, human hair wigs are typically fairly comfortable and last longer than acrylic wigs but commonly cost >£1,000,^27^ while acrylic wigs cost £70–£300 and often cause itching and overheating.^28^

Research also suggests a complex picture whereby wig‐wearing reduces fear of negative evaluation while simultaneously creating anxiety of being “found out” for wearing a wig.^4^ This may extend to other aesthetic products such as medical tattoos, hair systems and eyebrow transfers. Unmet expectations from products marketed at hair regrowth (such as shampoos^29^), and even medical treatment where results vary greatly,^30^ may also account for actively negative personal experiences. Further qualitative work would help to understand which products and services individuals find helpful, why, and the role of financial burden on their attributions.

Regarding gender and product use, although men used comparably fewer wigs and other aesthetic products aimed at concealing or camouflaging AA‐related hair loss, they did access products and services aimed at treatment and regrowth at similar rates, especially private dermatology. Accordingly, men with AA have reported wig‐wearing as socially undesirable, including holding comical associations,^11^ and other aesthetic techniques such as eyebrow microblading are likely to be interpreted as feminine.^16^ Equally, the coping literature suggests men are more likely to employ instrumental coping strategies during stressful health events than women,^31^ which may explain why men appeared more likely to seek out medical answers than aesthetic products.

Limitations of the study include the lack of representativeness and generalisability of the sample. In addition to Asian ethnicity, black individuals and men were underrepresented. The sampling methods also favoured those who are engaged in alopecia support and/or awareness‐raising and may underrepresent people with minimal heath service contact. The sample's skew towards higher severity in scalp hair loss may also not reflect the overall AA population and could potentially have attenuated scalp hair loss as a predictor of financial burden. Recording income bands rather than precise salaries may also have introduced measurement error in individual income values.

To conclude, this study suggests that women, those with high global AA symptom severity, and people with lower household incomes experience elevated financial burden from managing AA. These risk factors require consideration by health providers, commissioners, and policy makers when designing services to support the wellbeing of people living with AA.

## CONFLICT OF INTEREST STATEMENT

Dr Matthew Harries was Principal Investigator on the Soterios (Manentia) clinical trial for alopecia areata; He has previously held consultancy roles for Eli Lilly and Pfizer (paid to his institution). His consultancy on the current project was gifted to Alopecia UK. Dr Andrew Messenger was a collaborator on the Soterios (Manentia) clinical trial for alopecia areata. He has previously held consultancy roles for Pfizer. Dr Kerry Montgomery was awarded the grant from Pfizer as Principal Investigator and member of Alopecia UK to conduct the current study. Dr Fabio Zucchelli was contracted by Alopecia UK as an independent academic researcher for the current study. Pfizer had no involvement nor consultation in any of the study design, data collection, data analysis and manuscript preparation.

## AUTHOR CONTRIBUTIONS

**Fabio Zucchelli**: Conceptualization (supporting); data curation (equal); formal analysis (lead); funding acquisition (supporting); investigation (equal); methodology (equal); project administration (supporting); writing—original draft (lead); writing—review and editing (lead). **Matthew Harries**: Conceptualization (supporting); funding acquisition (supporting); methodology (supporting); supervision (equal); writing—review and editing (supporting). **Andrew Messenger**: Conceptualisation (supporting); funding acquisition (supporting); supervision (equal); writing—review and editing (supporting). **Kerry Montgomery**: Conceptualization (lead); funding acquisition (lead); investigation (equal); methodology (equal); project administration (lead); supervision (equal); writing—original draft (supporting); writing—review and editing (supporting).

## ETHICS STATEMENT

We gained ethical approval for the study from the Faculty Research Ethics Committee at UWE Bristol, on 08 April 2022 (reference HAS.21.09.009). Participants were presented with participant information (including a privacy statement) on Qualtrics followed by an itemised consent checklist, prior to taking the survey.

## Supporting information

Table S1

## Data Availability

The data that support the findings of this study are available on request from the corresponding author. The data are not publicly available due to privacy or ethical restrictions.
