# Supplementary material for: Establishing the financial burden of alopecia areata and its predictors
Source: Skin Health Dis. 2023 Oct 15;4(1):e301. doi: 10.1002/ski2.301 (PMC10831534; doi:10.1002/ski2.301)
Supplement: Supplementary file 1 — Table S1 [file SKI2-4-e301-s001.docx]

**Supplementary Table S1.** Breakdown of participants’ costs by category of products and services.

| Category of cost source | All participants (including non-spenders) | | Participants who spent in past 12 months | | | |
| --- | --- | --- | --- | --- | --- | --- |
|  |  |  | n (%*) | Median spend | | |
|  | Mean spend (SD) | Median |  | From all  spenders | From female spenders | From male  spenders |
| Wigs | £661.25 (£1133.30) | £150 | 449 (56.9%) | £700 | £700 | £50 |
| Headwear | £176.43 (£566.52) | £30 | 500 (63.9%) | £70 | £70 | £60 |
| Eyelash & eyebrow products | £151.68 (£254.16) | £30 | 457 (58.7%) | £200 | £200 | £260 |
| Other products | £181.81 (£634.51) | £20 | 440 (56.8%) | £120 | £120 | £115 |
| Prescriptions | £48.19 (££262.58) | £0 | 100 (12.9%) | £130.86 | £123.90 | £240 |
| Private Dermatology | £59.74 (£291.10) | £0 | 67 (8.7%) | £400 | £350 | £550 |
| Hair specialists | £48.91 (£343.66) | £0 | 60 (7.8%) | £200 | £200 | £200 |
| Health insurance claim costs | £6.47 (£68.06) | £0 | 14 (1.8%) | £220 | £195 | £725 |
| Private mental health services | £59.64 (£301.24) | £0 | 62 (8.0%) | £490 | £490 | £450 |
| Travel to appointments | £25.10 (£126.83) | £0 | 208 (26.8%) | £50 | £50 | £40 |
| Extra services (e.g. childcare) to cover appointments | £3.87 (£32.98) | £0 | 22 (2.8%) | £80 | £55 | £100 |
| PARTICIPANT’S TOTAL SPEND – from the sum of participants’ individual spending estimates | | | | | | |
| All sources of cost combined | £1481.66 (£2059.30) | £800  (Quartiles = £206-1941) | 774 (93.5%) | £840  (Quartiles = £290 - 1929) | £930  (Quartiles = £334 - 2050) | £250  (Quartiles = £60 - £850) |

*Total percentage exceeds 100 because participants were able to select multiple products and services. The reported percentages are taken from the total number of participants who responded to the pertinent question.
